# Child feeding practices and nutritional status of children aged 6–23 months in Nigeria: a multi-site survey

**DOI:** 10.1186/s12887-026-07075-z

**Published:** 2026-06-04

**Authors:** Gideon Onyedikachi Iheme, Idris Hajara, Adeleke Folasade Adebare, Linda Obianuju Edafioghor, Beulah Favour Ortutu, Chinaza Precious Uche, Tolulope Linder Owoyemi, Ifeoma Mercy Egechizuorom, Emmanuel Aanuoluwapo Oyebamiji, Obinna Chiemela Ogbonna

**Affiliations:** 1 Department of Food Studies, Nutrition and Dietetics, Uppsala University, Uppsala, SE-751 22 Sweden; 2 Department of Human Nutrition and Dietetics, Michael Okpara University of Agriculture, Umudike, Nigeria; 3 Department of Pediatrics, Federal Teaching Hospital Katsina, Katsina, Nigeria; 4 Department of Nutrition and Dietetics, Mobibbo Adama University Teaching Hospital, Yola, Nigeria; 5 Department of Nutrition and Dietetics, Federal University of Medical and Health Sciences Kwale, Kwale, Nigeria; 6 School of Food Science and Nutrition, University of Leeds, Leeds, UK; 7 Department of Human Nutrition and Dietetics, University of Ibadan, Ibadan, Nigeria; 8 Department of Nutrition and Dietetics, Federal Medical Centre, Umuahia, Nigeria; 9 Department of Dietetics and Human Nutrition, University of KwaZulu- Natal, Pietermaritzburg, South Africa; 10 Department of Nutrition and Dietetics, Obafemi Awolowo University Teaching Hospital, Ile-Ife, Nigeria

**Keywords:** Infant and young child feeding, Complementary feeding, Breastfeeding, Nutritional status, Childhood, Nigeria

## Abstract

**Background:**

Poor nutrition during early childhood affects growth/development and increases the risk of morbidity and mortality. This study assessed child feeding practices and the nutritional status of children from selected Nigerian tertiary health institutions.

**Methods:**

This study employed a descriptive cross-sectional design to digitally elicit responses from 1,295 mothers and child pairs accessing welfare services at four tertiary health institutions in Nigeria. Harmonized indicators such as Infant and Young Child Feeding Index (ICFI), WHO/UNICEF Infant and Young Child Feeding (IYCF) Practices Indicators, and WHO growth standards were used to evaluate the children’s feeding practices and anthropometric status. All descriptive and bivariate analysis (Fisher-Exact test) were done using IBM SPSS for Windows 25.

**Results:**

The study revealed that while 80.7% and 86.9% of the children met the minimum meal frequency and age-appropriate complementary feeding introduction criteria, breastfeeding initiation (48.5%), exclusive breastfeeding (EBF) rates (68.4%) and diversified food intake were suboptimal. However, 44.1% of children with stunting and 44.1% of children with overweight/obesity were identified. Exclusive breastfeeding was positively associated with BMI-for-age (OR = 1.51; 95% CI: 1.06–2.16; *p* = 0.02). Current breastfeeding status was also significantly associated with height-for-age (OR = 0.54; 95% CI: 0.37–0.78; *p* < 0.001) and BMI-for-age (OR = 1.78; 95% CI: 1.23–2.58; *p* = 0.002).

**Conclusions:**

Several aspects of the children’s feeding practices were sub-optimal, nutritional status was polarized, with many children affected by stunting and overweight/obesity. Interventions tailored towards strengthening breastfeeding and complementary feeding practices are critical for improving nutrition outcomes.

**Supplementary Information:**

The online version contains supplementary material available at 10.1186/s12887-026-07075-z.

## Background

Breastfeeding supports optimal growth and development while reducing disease risk in infants and young children [1–3]. It is recommended that breastfeeding be initiated within the first hour of life, given exclusively to the child throughout the first six months of life, and continued up to 2 years of age alongside complementary foods [4].

The complementary feeding period (6–23 months) is a critical period for growth and development in children, which requires special attention and care from mothers and caregivers. Malnutrition at this phase of life does not only cause immediate detrimental effects (such as increased infections, i.e., diarrhoea) but also predisposes a child to risks of stunting and increased chances of having cardiovascular diseases later in life, and death [5]. Given the significant influence that inadequate nutrition during the first 1000 days of life can have on children’s health and survival [5, 6], appropriate child-feeding practices have become an essential topic in public health discussions.

Complementary feeding refers to introducing appropriate soft, semi-solid, or solid foods to infants and young children alongside breastfeeding, starting at six months of age, to meet their nutritional needs [7]. Children under two years of age must receive safe, timely, adequate, and nutritious complementary foods [7]. Depending on their age, children should be offered food from at least five food groups daily [8]. The quality and timing of complementary feeding are also critical factors for ensuring optimal growth and development [9].

According to the United Nations Children’s Fund’s (UNICEF) 2024 report, one in three African children is poorly fed, and Nigeria is among the nations bearing the most significant burden [10]. In Nigeria, over a quarter (29%) of children under six months were exclusively breastfed. However, among children aged 6–23 months, only 12% of them were fed a minimum recommended diverse diet, culminating in just 6% of children meeting the minimum acceptable diet once age-appropriate meal frequency thresholds were considered [11]. Furthermore, many caregivers of children under five still struggle with understanding the importance of proper complementary feeding practices [12, 13]. For some, the challenge is not just about knowledge, nutritious diets are often out of reach for many families [14, 15]. As a result, children are frequently fed repetitive diets that provide energy but lack the essential nutrients needed for their growth and development [12, 14–16]. Thus, socio-economic inequalities and broader contextual factors such as politics, economy and the environment continue to pose threat to optimal complementary feeding practices which invariably affect nutritional outcomes, with their impact more profound in low-income settings than in developed climes [17–20].

Globally, approximately 50% of deaths among young children are attributed to poor nutrition [10, 21], with 80% of these deaths occurring in Sub-Saharan Africa [22]. Poor nutrition during early childhood deprives children of the opportunity to reach their full potential and increases their risk of dying before their fifth birthday [23]. Poor feeding practices have been linked to adverse health and nutritional outcomes [24]. The recent demographic and health survey presents more worrisome statistics; a good number of children under five were affected by stunting (40%), underweight (27%), and wasting (8%), with 1% being overweight or obese.

Malnutrition is not just a health issue but a development crisis with economic and social consequences. It is one of the leading causes of child deaths in Nigeria and deserves attention. While evidence for complementary feeding continues to expand in Nigeria [25], limited studies have looked at multiple indicators standardized for assessing infant and young child feeding (IYCF) and their implications on nutritional status. Therefore, to close this gap and assist relevant stakeholders with better evidence to support their children’s health and future, this study aims to demonstrate the association between multiple infant and young child feeding indicators and nutritional status.

## Methods

### Study design

This facility-based study is cross-sectional and descriptive in design.

### Study settings

This study was conducted across four tertiary hospitals in Nigeria: Federal Medical Center Umuahia in Abia State, Moddibo Adama University Teaching Hospital Yola in Adamawa State, Alex Ekwueme Federal University Teaching Hospital Abakaliki in Ebonyi State, and Federal Teaching Hospital Katsina in Katsina State. These tertiary institutions have a dedicated facility/centre called the Institute of Child Health Clinic and/or Child Outpatient Clinic, renowned for improving child health and reducing under-5 mortality.

These tertiary health institutions are situated within the municipal city centers where private sector employment (trading, artisanal jobs and service occupation), public sector jobs and farming or other agriculture-related activities represent the dominant sources of livelihood [26]. Umuahia and Abakaliki is characterized by a derived Savannah agroecological zone which is comparatively more fertile and supports crop-based farming system, comprising mainly of root crops (such as yam, coco-yam) and cereals (like maize rice) and vegetables. Conversely, selected locations (Yola and Katsina) situated within the Sudan Savannah operate an agro-pastoral system dominant by drought-tolerant crops like millet, sorghum, cowpea etc. and ruminant animals [27].

### Sampling and sampling techniques

About 20–30% of eligible mother-child pairs were selected using a simple random sampling (balloting without replacement) technique from lists of mothers who attended the facility on routine clinic days for welfare services during the survey period spanning February to April, 2024. This proportion culminated in a sample size deemed sufficient to detect small to moderate associations in multivariable analysis. A post-hoc power analysis, based on a total sample of *n* = 1,295 and α level of 0.05, demonstrated statistical power greater than 0.99 for both small (Cohen’s f² = 0.02) and moderate (f² = 0.15) effect sizes.

### Data collection procedures

#### Maternal socio-demographic characteristics and child feeding practices

Information on maternal socio-demographic characteristics, child-specific characteristics, and infant-young child feeding practices was collected using a structured questionnaire, with infant-young child feeding practices culled from the Infant and Child Feeding Index (ICFI) [28] and the WHO/UNICEF Infant and Young Child Feeding (IYCF) Practices Indicators [29].

This data collection process was conducted digitally using a web-based secure platform, Open Data Kit. The survey links were forwarded to mothers capable of independently completing the form with support by research assistants provided upon request. Those who could not complete the form independently, either lacking digital resources, were provided with mobile tablets or printed questionnaire copies, or facing literacy challenges were interviewed by trained research assistants. The selected participants were based on the following eligibility criteria.

Inclusion Criteria:

- Mothers of children aged six to twenty-three months attending the selected paediatrics clinics who gave their consent to participate in the study.
- Children aged six to twenty-three months who are apparently healthy, with no known chronic conditions (e.g., sickle cell disease, congenital disorders) and no recent acute illness (e.g., diarrhea, fever, or respiratory infections) in the preceding month.

Exclusion Criteria.

- Mothers with children under two years of age, with a chronic disease condition or recent acute illness, or did not give their consent.

### Anthropometric measurement of children

Anthropometric indices were employed to assess the nutritional status of the children. Participants were assigned identification numbers to input and match anthropometric values to appropriate background and complementary feeding information. Data on age (date of birth), length, and weight were collected/assessed in accordance with the stipulated guidelines by the World Health Organization (WHO) [30].

a] *Weight measurement*: The infants and children under two years old were weighed in light clothing, standing on a portable bathroom scale (New Weighing Scale Digital Bathroom Body Scale USB-Black, Model AD26). The scale was standardized with a known weight and set to zero before taking any measurements. The readings were taken to the nearest 0.1 kg.
b] *Height/Length measurement*: A stadiometer (Mechanical Stadiometer, Model SECA 213) was used to measure the older children’s height, which was read to the nearest 0.1 cm. The length board (Foldable Infantometer, SECA 417) was used to measure the younger babies’ length. Each child was laid on the board on a flat surface, and the length was also measured to the nearest 0.1 cm.

### Data analysis

#### Survey response rate and missing data

Responses were obtained from 1,295 mother–child pairs. However, the analytic sample varied across analyses due to differences in survey completeness. Specifically, between 919 and 1,295 observations were available for the infant and young child feeding (IYCF) practices module, while anthropometric data and infant/child feeding index were collected for 685 and 486 children, respectively. Data relevant to child anthropometric status and the infant and child feeding index were not collected at Alex Ekwueme Federal University Teaching Hospital Abakaliki due to logistical constraints related to the unavailability of measurement instruments.

Thus, the listwise deletion of observations with one or more missing variables required for index construction or for examining association between child feeding practices and anthropometric status implied that smaller analytic samples were used in the corresponding analysis. The Little MCAR test for continuous variables showed that missingness within the observed dataset is completely at random (p *≥* 0.05). However, the adjusted analyses based on the minimum complete responses are provided in Supplementary File 1 for reference.

### Infant and young child feeding practices

Information on Infant and Young Child Feeding practices was culled from two standardized indicators: Infant and Child Feeding Index (ICFI) [28] and the WHO/UNICEF Infant and Young Child Feeding (IYCF) Practices Indicators [29]. These indicators covered early initiation, exclusive and continued breastfeeding practices, bottle feeding, timely complementary feeding introduction, age-specific complementary feeding frequency, and diversity [28, 29]. Although the study population comprised children aged 6–23 months, caregiver recall was employed to capture earlier infant feeding practices, including all breastfeeding indicators and the introduction of semi-solid foods (for children over 8 months), while other indicators were calculated using age-appropriate denominators.

Specifically, ICF scores were derived from breastfeeding, avoidance of bottle feeding, meal frequency, age-appropriate feeding and dietary diversity information reported by the mothers during the interview. This yielded a possible maximum score of 12 points, categorized into low (1–4 points), moderate (5–8 points), and high (9–12 points) [28].

### Anthropometric status of children aged 6–23 months

The analysis used WHO Anthro 2006 v.3.2.2 software [31]. The Z-score was used to categorize respondents’ anthropometric status [31]. This classification applies to weight for age Z score (WAZ), Weight for length/height Z score (WHZ), length for age Z score (HAZ) and body mass index for age Z score (BMIAZ) indices.

Computations were based on the reference median recommended by the World Health Organization (WHO) and classified according to standard deviation units (Z-scores) based on WHO criteria [31, 32].

### Ethical approval and informed consent

Ethical approval was obtained from the Health and Research Ethics committee of the selected tertiary health institutions: Federal Medical Centre, Umuahia (FMC/QEH/G.596/Vol.10/576), Modibbo Adama University Teaching Hospital Yola (FMCY/SUB/S.128/161), Alex Ekwueme Federal University Teaching Hospital Abakaliki (AE-FUTHA/REC/VOL.3/2022/002), and Federal Teaching Hospital, Katsina (FTHKNREC.REG.25/06/22c/017). Written or digital informed consent was obtained from the respondents, and numbers were used to identify each respondent to ensure confidentiality. The respondents’ data was anonymized.

### Statistical analysis

Data from the sampled respondents was analyzed using SPSS for Windows 25 [33]. Socio-demographic and health-related characteristics, child feeding indicators, and anthropometric status of children under two years were first analyzed descriptively (mean, frequency, and percentage). Bivariate analysis was done using the Fisher-Exact test to assess the association between complementary feeding practices and nutritional status, with significance judged at *p* < 0.05. The sparse nature of datasets accruing from incomplete response rates makes the Fisher’s Exact test a suitable choice for exact probability calculation, thus maintaining the validity of statistical inference [34, 35].

## Results

### Maternal socio-demographic characteristics and child personal characteristics

A total of 1,295 mothers of children under two years were interviewed across four tertiary health institutions in Nigeria. The majority (78.8%) of the women interviewed were aged 19–39 years. Most (60.8%) of the mothers attained tertiary education. A good number of the mothers were civil or public servants (38.9%), while 23% and 16.4% were housewives and traders, respectively. The mean age of the children in the study was 11.44 months (S.D. ± 7.34), with a gender distribution of 48.3% female and 51.7% male. (Suppl. File 1).

### Infant and young child feeding practices

Table 1 reports the infant and young child feeding practices, about one-tenth (8.1% of the males and 13.1% of the females) were not breastfed. Breastfeeding was initiated within an hour of birth for close to half (48% of males and 48.1% of females) of the respondents. Exclusive breastfeeding was practiced by 68.4% of the mothers, with significantly (*p* < 0.05) more female (71.3%) than male (65.9%) children as beneficiaries. Likewise, 44.4% of the respondents indicated using bottles to feed their children, similar spread across both genders (43.9% of males and 44.9% of females). The majority (86.9%) were introduced to semi-solid foods at an appropriate age. After six months, more than half (59.9%) of the mothers feed their children more than five times daily. This resulted in majority (80.7%) of the children meeting the minimum frequency of meals appropriate for their age, varying slightly but significantly) *p* < 0.05) across sexes (78.7 – males; 83.1% - females).

**Table 1:** Gender Distribution of Infant and Young Child Feeding Practices

Variables	Male		Female		Total		*p*-value
	*N*	%	*N*	%	*N*	%	
Child ever breastfed							0.002^*^
No	54	8.1	82	13.1	136	10.5	
Yes	616	91.9	543	86.9	1159	89.5	
Initiation of breastfeeding							0.859
Within an hour of birth	259	48.8	216	48.1	476	48.5	
2–12 h	145	27.3	124	27.5	269	27.4	
12–24 h	80	15.1	72	16	152	15.5	
3–5 days	39	7.3	27	6	66	6.7	
> 5days	8	1.5	11	2.4	19	1.9	
Practiced exclusive breastfeeding							0.042^*^
No	180	34.1	129	28.7	309	31.6	
Yes	348	65.9	320	71.3	668	68.4	
Introduced semi-solid food to child within 6–8 months of age							0.488
No	67	13	59	13.2	126	13.1	
Yes	450	87	387	86.8	837	86.9	
Child was fed from a nipple-shaped bottle							0.398
No	284	56.1	239	55.1	523	55.6	
Yes	222	43.9	195	44.9	417	44.4	
No of times children were fed CF in 24 h before survey							0.675
Once	39	6.4	34	6	73	6.2	
Twice	45	7.4	44	7.7	89	7.5	
Three times	175	28.7	147	25.8	2	27.3	
Four times	166	27.2	178	31.2	344	29.2	
> 5 times	185	30.3	167	29.3	352	29.8	
Minimum Meal Frequency							0.023^*^
Not Met	105	21.3	72	16.9	177	19.3	
Met	387	78.7	355	83.1	742	80.7	

### Food consumption over 24 hours before interview

**Fig. 1:**
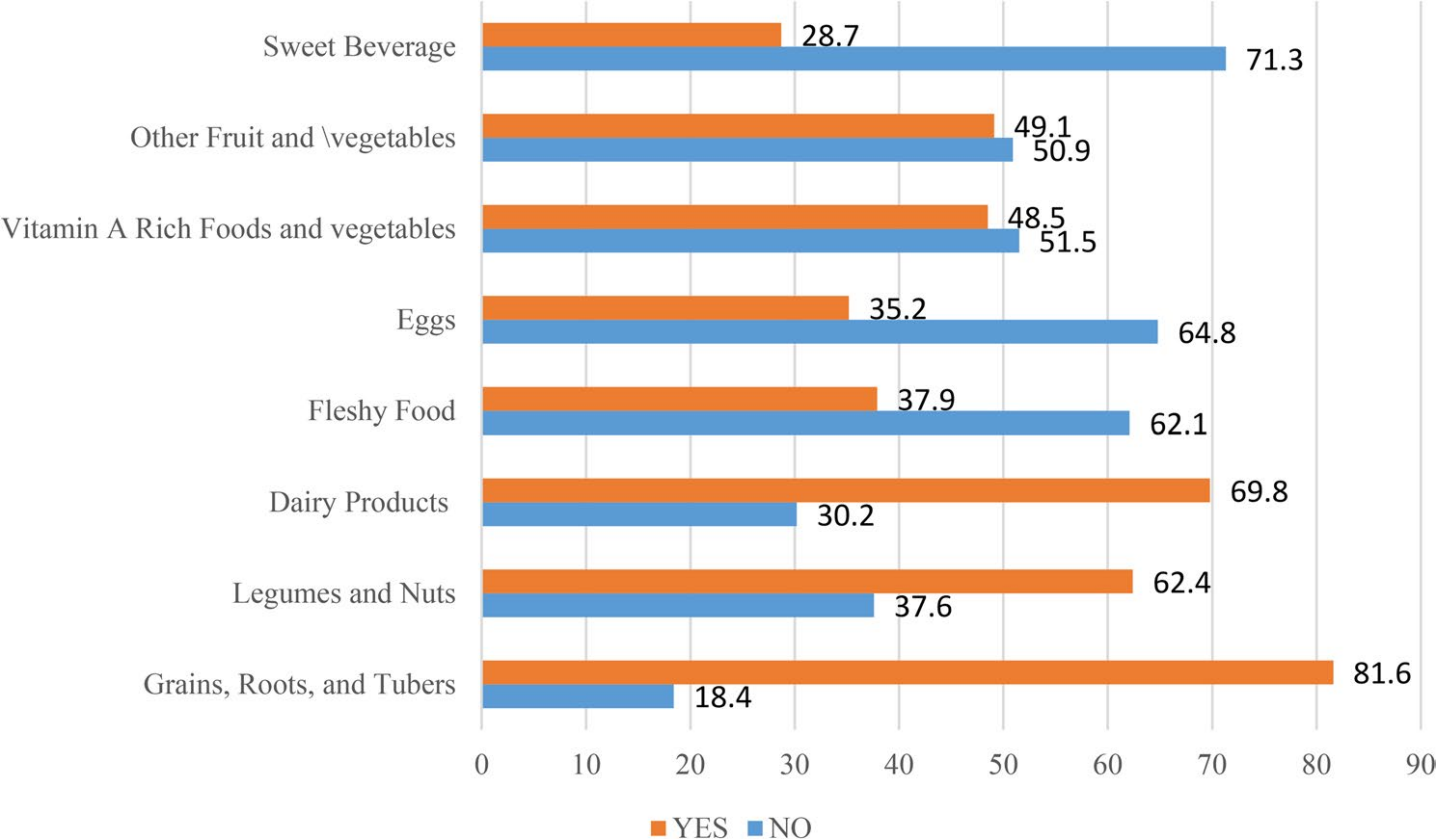
Types of food groups consumed by infants in the previous 24hours

The figure below shows the frequency of consumption of food groups by the children over twenty-four hours before the survey. Compared to other food groups mentioned, most of the children were fed foods within the starchy staples (81.6%), pulses (62.4%), and dairy products (69.8) categories. About half of the children aged 6–23 months consumed Vitamin A rich foods and vegetables (48.5%), as well as other fruits and vegetables (49.1%), with eggs (35.2%) and fleshy foods (37.9%) and sweet beverages (28.7%) receiving lower intake proportion (Fig. 1).

### Infant child feeding index score

The infant child feeding index score, as described by Ruel, is indicated in (Table 2).

**Table 2:** RUEL Infants and Child Feeding Index Score

RUEL Scores (*n* = 486)	Male		Female		Total		*p*-value
	*N*	%	*N*	%	*N*	%	
							0.168
Low (1–4)	30	11.7	40	17.4	70	14.4	
Moderate (5–8)	176	68.8	143	62.2	319	65.6	
High (9–12)	50	19.5	47	20.4	97	20	
Mean ± SD	6.66 ± 1.94		6.54 ± 2.09		6.60 ± 2.01		

ICFI scores were computed based on individual breastfeeding (0–2 points), avoidance of bottle feeding (0–1 point), meal frequency (0–2 points), dietary diversity (0–4 points), and age-appropriate feeding (0–3 points) indicators

This yielded a composite score of 12, categorized as low (<4 points), medium (5-8 points) and high (9-12 points)

Based on RUEL’s infant and child feeding index scores, only 20% of children aged 6–23 months comprising 19.5% of males and 20.4% females had high ICFI level, while the majority (65.6%) were classified within the moderate category. Only 14.4% of the respondents had low ICFI scores, comprising 11.7% for males and 17.4% for females.

### Nutritional status of the children in the study

The nutritional status of the children is indicated in (Table 3). Overall, the prevalence of wasting, stunting and overweight/obesity was substantially high. Less than 10% of the respondents were affected by wasting, with a slightly higher proportion of females (9.7%) compared to males (8.5%). Close to half of the children experienced stunting (44.1%), which was almost evenly disaggregated across male (45.7%) and females (42.1%). Being affected by underweight accounted for 12.3% of the study population, with 13.3% of males and 11.0% of female respondents affected. In contrast, persons with overweight and obesity were more prevalent, affecting 44.1% of the respondents.

**Table 3:** Nutritional Status of Children by Gender

Variables	Male		Female		Total		*p*-value	OR (95% CL)
	*N*	%	*N*	%	*N*	%		
WHZ							0.335	0.86 (0.51–1.45)
Affected by wasting	32	8.5	30	9.7	62	9.1		
Normal	344	91.5	279	90.3	623	90.9		
HAZ							0.188	1.16 (0.86–1.57)
Affected by Stunting	172	45.7	130	42.1	302	44.1		
Normal	204	54.3	179	57.9	383	55.9		
WAZ							0.214	1.24 (0.78–1.97)
Affected by underweight	50	13.3	34	11	84	12.3		
Normal	326	86.7	275	89	601	87.7		
BMIAZ							0.517	0.99 (0.73–1.35)
Normal	210	55.9	173	56	383	55.9		
Affected by Overweight/obesity	166	44.1	136	44	302	44.1		

*WHZ* Weight for Length/Height, *HAZ* Length/Height for Age, *WAZ* Weight for Age, *BMIAZ* BMI for age

Nutritional status was classified as <-2 SD (under-nutrition: wasting, stunting, or underweight), -2 to +2 SD (normal), and >+2 SD (overweight/obesity)

*CI* confidence interval, *OR* odds ratio

### Association between nutritional status and WHO/UNICEF IYCF practices

The association between infant and young child feeding indicators and the nutritional status of the respondents is described in (Table 4). Overall, positive associations were observed across all nutritional status indicators, suggesting that adherence to recommended child feeding practices is associated with normal anthropometric outcomes. Exclusive breastfeeding was significantly associated with BMI-for-age (OR = 1.51; 95% CI: 1.06–2.16; *p* = 0.02). Current breastfeeding status was also significantly associated with height-for-age (OR = 0.54; 95% CI: 0.37–0.78; *p* < 0.001) and BMI-for-age (OR = 1.78; 95% CI: 1.23–2.58; *p* = 0.002). On the other hand, introduction of solid, semi-solid, or soft foods at 6–8 months was associated with BMI-for-age (OR = 2.14; 95% CI: 0.98–4.36; *p* = 0.03), although the confidence interval included the null value. Consumption of fleshy foods, eggs, vitamin A-rich foods, and sweet beverages was not significantly associated with any nutritional status indicators.

**Table 4:** Association between nutritional status and infant/young child feeding practices

Variables	WHZ		*p*-value		HAZ		*p*-value		WAZ		*p*-value		BMIAZ		*p*-value	
	Affected by Wasting *N* (%)	Normal *N* (%)		OR (95% CL)	Affected by Stunting *N* (%)	Normal *N* (%)		OR (95% CL)	Affected by Underweight *N* (%)	Normal *N* (%)		OR (95% CL)	Normal *N* (%)	Overweight/ Obesity *N* (%)		OR (95% CL)
Early Initiation of Breastfeeding			0.20	1.47 (0.76–2.73)			0.26	0.79 (0.53–1.17)			0.15	1.50 (0.85–2.57)			0.13	1.36 (0.92–2.02)
No	17 [27.4)	127 [20.5)			57 (18.9)	87 (22.7)			23 (27.4)	121 (20.1)			89 (23.2)	55 (18.2)		
Yes	45 (72.6)	495 (79.6)			245 (81.1)	296 (77.3)			61 (72.6)	480 (79.9)			294 (76.8)	247 (81.8)		
Practiced exclusive breastfeeding			0.06	1.71 (0.95–3.03)			0.67	0.93 (0.65–1.31)			0.30	1.32 (0.78–2.20)			0.02^*^	1.51 (1.06–2.16)
No	24 (39.3)	170 (27.5)			83 (27.7)	111 (29.2)			28 (33.7)	166 (27.8)			122 (32.2)	72 (23.9)		
Yes	37 (60.7)	448 (72.5)			217 (72.3)	269 (70.8)			55 (66.3)	431 (72.2)			257 (67.8)	229 (76.1)		
Currently breastfeeding child			0.23	1.43 (0.78–2.55)			< 0.001^***^	0.54 (0.37–0.78)			0.08	0.60 (0.32–1.08)			0.002^**^	1.78 (1.23–2.58)
No	21 (33.9)	162 (26.4)			61 (20.5)	122 (32.3)			16 (19)	167 (28.2)			121 (31.9)	62 (20.9)		
Yes	41 (66.1)	451 (73.6)			237 (79.5)	256 (67.7)			68 (81)	425 (71.8)			258 (68.1)	235 (79.1)		
Introduced solid, semi-solid or soft foods to child at 6–8 months			0.46	0.55 (0.11–1.78)			0.40	1.28 (0.71–2.29)			0.03^*^	2.14 (0.98–4.36)			0.09	0.61 (0.34–1.10)
No	3 (4.9)	52 (8.6)			28 (9.5)	28 (7.6)			12 (14.8)	44 (7.5)			25 (6.7)	31 (10.5)		
Yes	58 (95.1)	552 (91.4)			268 (90.5)	342 (92.4)			69 (85.2)	541 (92.5)			347 (93.3)	263 (89.5)		
Fleshy food in the past 24 h			1	1.11 (0.43–3.03)			0.65	0.84 (0.45–1.58)			0.63	1.21 (0.62–2.43)			0.51	0.78 (0.38–1.59)
No	16 (64.0)	101 (61.6)			55 (59.8)	62 (63.9)			39 (65.0)	78 (60.5)			82 (60.3)	35 (66.0)		
Yes	9 (36.0)	63 (38.4)			37 (40.2)	35 (36.1)			21 (35.0)	51 (39.5)			54 (39.7)	18 (34.0)		
Eggs in the past 24 h			0.80	1.33 (0.45–4.83)			0.74	1.17 (0.57–2.42)			0.59	1.24 (0.57–2.82)			0.45	1.34 (0.60–2.89)
No	20 (80.0)	123 (75.0)			71 (77.2)	72 (74.2)			47 (78.3)	96 (74.4)			105 (77.2)	38 (71.7)		
Yes	5 (20.0)	41 (25.0)			21 (22.8)	25 (25.8)			13 (21.7)	33 (25.6)			31 (22.8)	15 (28.3)		
Vitamin A rich foods and vegetables in the past 24 h			0.65	1.37 (0.51– 4.11)			0.76	1.12 (0.58–2.13)			0. 10	1.83 (0.89–3.93)			0.74	1.13 (0.40–3.67)
No	18 (72.0)	107 (65.2)			62 (67.4)	63 (64.9)			45 (75.0)	80 (62.0)			91 (66.9)	34 (64.2)		
Yes	7 (28.0)	57 (34.8)			30 (32.6)	34 (35.1)			15 (25.0)	49 (38.0)			45 (33.1)	19 (35.8)		
Sweet beverage in the past 24 h			1	1.13 (0.40–3.67)			0.74	0.88 (0.44–1.78)			0.86	0.95 (0.45–2.04)			0.58	0.78 (0.34–1.73)
No	19 (76.0)	121 (73.8)			67 (72.8)	73 (75.3)			44 (73.3)	96 (74.4)			99 (72.8)	41 (77.4)		
Yes	6 (24.0)	43 (26.2)			25 (27.2)	24 (24.7)			16 (26.7)	33 (25.6)			37 (27.2)	12 (22.6)		

^***^ Statistically significant at *P* < 0.001; ^**^ Statistically significant at *P* < 0.01; ^*^ Statistically significant at *P* < 0.05

Fisher’s exact test; *CI* confidence interval, *OR* odds ratio

## Discussion

This study provides valuable insights into maternal child feeding habits and how they impact their nutritional outcomes in four tertiary health institutions across Nigeria. A considerable proportion of mothers in this study (60.8%) had tertiary education, reflecting the urban environment which exceeded national reports of 13.7% women of reproductive age with a post-secondary educational qualification [11]. Educated women seek healthcare more actively and adopt healthier practices, which is why maternal education is linked to improved child health outcomes [3]. The predominance of women aged 19–29 years in this study closely aligns with national data indicating a median maternal age of 21.3 years at first childbirth, as well as evidence that approximately three-quarters of women of reproductive age have had a live birth by age 25 [11]. The mix of working mothers and those engaged in domestic work reflects the diverse socioeconomic status of participants, a pattern observed in other urban areas in Africa [36, 37]. Likewise, the closely balanced participation of male and female children and a mean age of 11.44 months highlight the representativeness of the sample with respect to these child characteristics.

Compared with existing pooled evidence, the prevalence of early initiation of breastfeeding observed in this study was generally lower, whereas exclusive breastfeeding rates were higher [38–40]. Conversely, reported rates of introduction of semi-solid foods and minimum meal frequency were higher than the national averages [11]. These disparities may be attributed to methodological differences, particularly the use of caregiver recall of past feeding practices for some age-specific infant and young child feeding indicators.

The mean ICFI score of 6.60 ± 2.01 for the total group can be compared to the mean ICFI scores found by Çelik and Köksal in 2024 for different age groups [41]. The study found mean ICFI scores of 7.7 ± 1.9 in the 6-8-month group, 9.3 ± 1.6 in the 9-11-month group, and 9.10 ± 1.6 in the 12–24 months group. However, the absence of gender distribution in similar studies limited possible comparison. This low Infant and Child Feeding Index (ICFI) score when compared with other studies is reflective of overall poor nursing practices, infrequent feeding, and a lack of dietary diversity which are key contributors to chronic undernutrition [28, 29]. Thus, the low consumption of micronutrient-dense and protein-rich foods, such as fleshy foods, eggs, and fruits and vegetables observed in this study, warrants attention given their critical role in child growth and development [42–45]. In contrast, limiting the intake of sweetened beverages is encouraged due to their poor dietary quality and potential to displace nutrient-dense foods [46, 47].

The prevalence of wasting and stunting in this study was broadly comparable with national estimates [10]. However, other facility-based studies have reported higher rates of wasting (11.2–13.4%) and lower levels of stunting (20.8–28.4%) [48, 49]. In contrast, the present study observed a markedly lower prevalence of underweight (12.3%) and a substantially higher prevalence of overweight and obesity (44.1%) compared with previous reports, which documented underweight prevalence ranging from 18% to 27% and overweight/obesity between 2% and 28.4% [10, 48, 49]. These highlights the need for strengthening the sustained uptake of proven interventions that improve optimal feeding and care practices for children, while simultaneously addressing nutrition transition dynamics that are increasingly associated with rising rates of childhood overweight and obesity [50, 51]. Furthermore, evidence suggests that boys are more susceptible to undernutrition, as shown by a slightly higher prevalence of stunting in boys than girls [52]. However, the result shows no remarkable related gender differences in the report of wasting, stunting, underweight, and overweight/obesity among the respondents, and this is in agreement with Palacios et al. [53], , in the study on the factors associated with the nutritional status of infants and young children from rural Honduras, where no gender-related differences were reported for stunting, overweight, or wasting, but the same study reported that the prevalence of underweight were significantly lower in females having 71% lower odds of being underweight [53].

Delayed initiation is linked to both underweight and overweight conditions. Highlighting its protective benefits against malnutrition and excess weight, exclusive breastfeeding (EBF) was practised by an impressive 68.4% of mothers, significantly higher than the national average of 29%. This practice was also strongly associated with better weight-for-height (WHZ) and BMI-for-age (BMIAZ) outcomes [54].

Numerous expert bodies recommend optimal breastfeeding and safe, adequate complementary feeding practices as critical strategies for reducing the risk of all forms of malnutrition in children and their associated consequences [55–57]. Similar to study findings, exclusive and continued breastfeeding were associated with lower rates of stunting, underweight and overweight in other studies [58, 59]. The introduction of semi-solid foods was associated with normal weight-related status, including weight-for-age and BMI-for-age. This finding is consistent with evidence from previous systematic reviews, which indicate that early introduction of complementary foods is associated with an increased risk of overweight and obesity, and in some instances, underweight [42, 60, 61].

### Limitations of the study

The infant and young child feeding practices in the study rely on recall by the children’s mothers/caregivers, which may be prone to bias, particularly for age-specific indicators that fall outside the study population’s age range. However, the children’s dietary intake and anthropometry were assessed twice to validate the information provided or measured.

While digital data collection of feeding practices has proven efficient and cost-effective, it is typically marred by low or incomplete response rates, which account for the varying response rates reported in this study.

## Conclusion

Based on the findings of this study, which assessed child feeding practices and nutritional status among children aged 6–23 months across four health facilities in Nigeria. This study revealed a mixed picture of IYCF practices in Nigeria. While a significant proportion of mothers met the minimum meal frequency, early breastfeeding initiation, and exclusive breastfeeding rates, food consumption diversity was suboptimal. However, this study incorporated gender distribution into its reporting of IYCF practices, showing slight differences in breastfeeding initiation, exclusive breastfeeding, and minimum meal frequency between males and females.

Nutritional Status information demonstrated the co-existence of substantial levels of stunting and overweight/obesity in this study. Child feeding practices such as exclusive breastfeeding, currently breastfeeding and timely introduction of semi-solid foods showed significant associations with various nutritional status indicators.

The findings of this study not only add to the existing body of knowledge on infant and young child feeding but also highlight the urgent need for improved nutritional education for mothers and caregivers, especially on early and exclusive breastfeeding, including the adequacy and diversity of complementary foods. Ultimately, these interventions are essential to improving the health and nutritional status of children aged under 24 months and reducing the burden of malnutrition in Nigeria.

## Supplementary Information

Supplementary Material 1.

## Data Availability

Data used in this study are available to the corresponding author and can be shared upon reasonable request.

## Abbreviations

UNICEF: United Nations Children's Fund's
MICS: Multiple Indicator Cluster Survey
IYCF: Infant and young child feeding
WHO: World Health Organization
WAZ: Weight for age Z score
WHZ: Weight for length/height Z score
HAZ: Height for age Z score
BMIAZ: Body mass index for age Z score
NDHS: Nigeria Demographic and Health Survey
EBF: Exclusive breastfeeding
ICFI: The Infant and Child Feeding Index
