# Supplementary material for: Child feeding practices and nutritional status of children aged 6–23 months in Nigeria: a multi-site survey
Source: BMC Pediatr. 2026 Jun 4;26:550. doi: 10.1186/s12887-026-07075-z (PMC13248362; doi:10.1186/s12887-026-07075-z)
Supplement: Supplementary file 1 — Supplementary Material 1. [file 12887_2026_7075_MOESM1_ESM.docx]

**Supplementary File 1: Complete-case adjusted analysis (n = 189)**

**Table 1: Gender Distribution of Infant and Young Child Feeding Practices (n = 189)**

| **Variables** | **Male** | | **Female** | | **Total** | | **p-value** |
| --- | --- | --- | --- | --- | --- | --- | --- |
|  | **N** | **%** | **N** | **%** | **N** | **%** |  |
| **Child ever breastfed** |  |  |  |  |  |  | 0.215 |
| No | 0 | 0 | 2 | 2.3 | 2 | 1.1 |  |
| Yes | 101 | 100 | 86 | 97.7 | 187 | 98.9 |  |
| **Initiation of breastfeeding** |  |  |  |  |  |  | 0.385 |
| Within an hour of birth | 56 | 55.4 | 51 | 58 | 107 | 56.6 |  |
| 2-12 hours | 21 | 20.8 | 20 | 22.7 | 41 | 21.7 |  |
| 12-24 hours | 11 | 10.9 | 13 | 14.8 | 24 | 12.7 |  |
| 3-5 days | 9 | 8.9 | 3 | 3.4 | 12 | 6.3 |  |
| >5days | 4 | 4 | 1 | 1.1 | 5 | 2.6 |  |
| **Practiced exclusive breastfeeding** |  |  |  |  |  |  | 0.543 |
| No | 39 | 39.4 | 34 | 39.1 | 73 | 39.2 |  |
| Yes | 60 | 60.6 | 53 | 60.9 | 113 | 60.8 |  |
| **Introduced semi-solid food to child**  **within 6-8 months of age** |  |  |  |  |  |  | 0.600 |
| No | 3 | 3.1 | 3 | 3.5 | 6 | 3.3 |  |
| Yes | 94 | 96.9 | 83 | 96.5 | 177 | 96.7 |  |
| **Child was fed from a nipple-shaped bottle** |  |  |  |  |  |  | 0.030^*^ |
| No | 46 | 47.9 | 27 | 32.9 | 73 | 41 |  |
| Yes | 50 | 52.1 | 55 | 67.1 | 105 | 59 |  |
| **No of times children were fed CF in 24hrs before** **survey** |  |  |  |  |  |  | 0.589 |
| Once | 7 | 7.4 | 9 | 10.7 | 16 | 8.9 |  |
| Twice | 12 | 12.6 | 12 | 14.3 | 24 | 13.4 |  |
| Three times | 29 | 30.5 | 27 | 32.1 | 56 | 31.3 |  |
| Four times | 18 | 18.9 | 19 | 22.6 | 37 | 20.7 |  |
| >5 times | 29 | 30.5 | 17 | 20.2 | 46 | 25.7 |  |

**Table 2. RUEL Infants and Child Feeding Index Score (n = 189)**

| **RUEL Scores (n= 189)** | **Male** | | **Female** | | **Total** | | **p-value** |
| --- | --- | --- | --- | --- | --- | --- | --- |
|  | **N** | **%** | **N** | **%** | **N** | **%** |  |
|  |  |  |  |  |  |  | 0.294 |
| Low (1-4) | 12 | 11.9 | 17 | 19.3 | 29 | 15.3 |  |
| Moderate (5-8) | 75 | 74.3 | 57 | 64.8 | 132 | 69.8 |  |
| High (9-12) | 14 | 13.9 | 14 | 15.9 | 28 | 14.8 |  |

**Table 3: Nutritional Status of Children by Gender (n = 189)**

| **Variables** | **Male** | | **Female** | | **Total** | | **p-value** | **OR (95% CL)** |
| --- | --- | --- | --- | --- | --- | --- | --- | --- |
|  | N | % | N | % | N | % |  |  |
| WHZ |  |  |  |  |  |  | 0.313 | 1.36 (0.58 – 3.21) |
| Affected by wasting | 15 | 14.9 | 10 | 11.4 | 25 | 13.2 |  |  |
| Normal | 86 | 85.1 | 78 | 88.6 | 164 | 86.8 |  |  |
|  |  |  |  |  |  |  |  |  |
| HAZ |  |  |  |  |  |  | 0.348 | 1.17 (0.66 – 2.07) |
| Affected by Stunting | 51 | 50.5 | 41 | 46.6 | 92 | 48.7 |  |  |
| Normal | 50 | 49.5 | 47 | 53.4 | 97 | 51.3 |  |  |
|  |  |  |  |  |  |  |  |  |
| WAZ |  |  |  |  |  |  | 0.327 | 1.21 (0.65 – 2.24) |
| Affected by underweight | 34 | 33.7 | 26 | 29.5 | 60 | 31.7 |  |  |
| Normal | 67 | 66.3 | 62 | 70.5 | 129 | 68.3 |  |  |
|  |  |  |  |  |  |  |  |  |
| BMIAZ |  |  |  |  |  |  | 0.478 | 0.93 (0.49 – 1.76) |
| Normal | 72 | 71.3 | 64 | 72.7 | 136 | 72 |  |  |
| Affected by Overweight/obesity | 29 | 28.7 | 24 | 27.3 | 53 | 28 |  |  |

**Table 4: Association between nutritional status and infant/young child feeding practices (n=189)**

| Variables | WHZ | | p-value |  | HAZ | | p-value |  | WAZ | | p-value |  | BMIAZ | | p-value |  |
| --- | --- | --- | --- | --- | --- | --- | --- | --- | --- | --- | --- | --- | --- | --- | --- | --- |
|  | **Affected by Wasting**  **N (%)** | **Normal**  **N (%)** |  | **OR (95% CL)** | **Affected by Stunting N (%)** | **Normal**  **N (%)** |  | **OR (95% CL)** | **Affected by Underweight N (%)** | **Normal**  **N (%)** |  | **OR (95% CL)** | **Normal**  **N (%)** | **Overweight/**  **Obesity**  **N (%)** |  | **OR (95% CL)** |
| Early Initiation of Breastfeeding |  |  |  |  |  |  |  |  |  |  |  |  |  |  |  |  |
| No |  |  |  |  |  |  |  |  |  |  |  |  |  |  |  |  |
| Yes |  |  |  |  |  |  |  |  |  |  |  |  |  |  |  |  |
|  |  |  |  |  |  |  |  |  |  |  |  |  |  |  |  |  |
| Practiced exclusive breastfeeding |  |  | 0.012 | 2.99 (1.23 – 7.25) |  |  | 0.103 | 0.65 (0.36 – 1.18) |  |  | 0.454 | 1.09 (0.58 – 2.05) |  |  | 0.002 | 2.90 (1.40 – 6.00) |
| No | 15 (62.5) | 58 (35.8) |  |  | 31 (34.1) | 42 (44.2) |  |  | 24 (40.7) | 49 (38.6) |  |  | 61 (45.9) | 12 (22.6) |  |  |
| Yes | 9 (37.5) | 104 (64.2) |  |  | 60 (65.9) | 53 (55.8) |  |  | 35 (59.3) | 78 (61.4) |  |  | 72 (54.1) | 41 (77.4) |  |  |
|  |  |  |  |  |  |  |  |  |  |  |  |  |  |  |  |  |
| Currently breastfeeding child |  |  | 0.311 | 1.38 (0.57 – 3.33) |  |  | 0.064 | 0.58 (0.31 – 1.10) |  |  | 0.117 | 0.62 (0.31 – 1.24) |  |  | 0.115 | 1.67 (0.80 – 3.49) |
| No | 9 (36) | 47 (29) |  |  | 22 (24.2) | 34 (35.4) |  |  | 14 (23.3) | 42 (33.1) |  |  | 44 (32.8) | 12 (22.6) |  |  |
| Yes | 16 (64) | 115 (71) |  |  | 69 (75.8) | 62 (64.6) |  |  | 46 (76.7) | 85 (66.9) |  |  | 90 (67.2) | 41 (77.4) |  |  |
|  |  |  |  |  |  |  |  |  |  |  |  |  |  |  |  |  |
| Introduced solid, semi-solid or soft foods to child at 6-8 months |  |  | 0.190 | 3.35 (0.58 – 19.32) |  |  | 0.326 | 2.12 (0.38 – 11.85) |  |  | 0.081 | 4.56 (0.81 – 25.63) |  |  | 0.234 | 0.39 (0.08 – 2.02) |
| No | 2 (8) | 4 (2.5) |  |  | 4 (4.4) | 2 (2.2) |  |  | 4 (6.9) | 2 (1.6) |  |  | 3 (2.3) | 3 (5.7) |  |  |
| Yes | 23 (92) | 154 (97.5) |  |  | 86 (95.6) | 91 (97.8) |  |  | 54 (93.1) | 123 (98.4) |  |  | 127 (97.7) | 50 (94.3) |  |  |
|  |  |  |  |  |  |  |  |  |  |  |  |  |  |  |  |  |
| Fleshy food in the past 24hrs |  |  | 0.501 | 1.11 (0.46 – 2.66) |  |  | 0.332 | 0.84 (0.47 – 1.51) |  |  | 0.333 | 1.21 (0.64 – 2.30) |  |  | 0.288 | 0.78 (0.40 – 1.52) |
| No | 16 (64) | 101 (61.6) |  |  | 55 (59.8) | 62 (63.9) |  |  | 39 (65) | 78 (60.5) |  |  | 52 (60.3) | 35 (66) |  |  |
| Yes | 9 (36) | 63 (38.4) |  |  | 37 (40.2) | 35 (36.1) |  |  | 21 (35) | 51 (39.5) |  |  |  |  |  |  |
|  |  |  |  |  |  |  |  |  |  |  |  |  |  |  |  |  |
| Eggs in the past 24hrs |  |  | 0.397 | 1.33 (0.47 – 3.78) |  |  | 0.382 | 1.17 (0.60 – 2.29) |  |  | 0.348 | 1.24 (0.60 – 2.58) |  |  | 0.270 | 1.34 (0.65 – 2.75) |
| No | 20 (80) | 123 (75) |  |  | 71 (77.2) | 72 (74.2) |  |  | 47 (78.3) | 96 (74.4) |  |  | 105 (77.2) | 38 (71.7) |  |  |
| Yes | 5 (20) | 41 (25) |  |  | 21 (22.8) | 25 (25.8) |  |  | 13 (21.7) | 33 (25.6) |  |  | 31 (22.8) | 15 (28.3) |  |  |
|  |  |  |  |  |  |  |  |  |  |  |  |  |  |  |  |  |
| Vitamin A rich foods and vegetables in the past 24hrs |  |  | 0.337 | 1.37 (0.54 – 3.47) |  |  | 0.421 | 1.12 (0.61 – 2.04) |  |  | 0.054 | 1.84 (0.93 – 3.64) |  |  | 0.422 | 1.13 (0.58 – 2.20) |
| No | 18 (72) | 107 (65.2) |  |  | 62 (67.4) | 63 (64.9) |  |  | 45 (75) | 80 (62) |  |  | 91 (66.9) | 34 (64.2) |  |  |
| Yes | 7 (28) | 57 (34.8) |  |  | 30 (32.6) | 34 (35.1) |  |  | 15 (25) | 49 (38) |  |  | 45 (33.1) | 19 (35.8) |  |  |
|  |  |  |  |  |  |  |  |  |  |  |  |  |  |  |  |  |
| Sweet beverage in the past 24hrs |  |  | 0.515 | 1.13 (0.42 – 3.00) |  |  | 0.415 | 0.88 (0.46 – 1.69) |  |  | 0.504 | 0.95 (0.47 – 1.90) |  |  | 0.327 | 0.78 (0.37 – 1.65) |
| No | 19 (76) | 121 (73.8) |  |  | 67 (72.8) | 73 (75.3) |  |  | 44 (73.3) | 96 (74.4) |  |  | 99 (72.8) | 41 (77.4) |  |  |
| Yes | 6 (24) | 43 (26.2) |  |  | 25 (27.2) | 24 (24.7) |  |  | 16 (26.7) | 33 (25.6) |  |  | 37 (27.2) | 12 (22.6) |  |  |
